# Emerging Roles of Protease-Activated Receptors (PARs) in the Modulation of Synaptic Transmission and Plasticity

**DOI:** 10.3390/ijms22020869

**Published:** 2021-01-16

**Authors:** Rachel Price, Nicola Biagio Mercuri, Ada Ledonne

**Affiliations:** 1 Department of Experimental Neuroscience, IRCCS Fondazione Santa Lucia, 00143 Rome, Italy; Pricerach89@gmail.com (R.P.); mercurin@med.uniroma2.it (N.B.M.); 2 Department of Systems Medicine, Università di Roma “Tor Vergata”, 00133 Rome, Italy

**Keywords:** protease-activated receptors, serine proteases, matrix metalloproteases, glutamate, GABA, synaptic transmission, synaptic plasticity

## Abstract

Protease-activated receptors (PARs) are a class of G protein-coupled receptors (GPCRs) with a unique mechanism of activation, prompted by a proteolytic cleavage in their N-terminal domain that uncovers a tethered ligand, which binds and stimulates the same receptor. PARs subtypes (PAR1-4) have well-documented roles in coagulation, hemostasis, and inflammation, and have been deeply investigated for their function in cellular survival/degeneration, while their roles in the brain in physiological conditions remain less appreciated. Here, we describe PARs’ effects in the modulation of neurotransmission and synaptic plasticity. Available evidence, mainly concerning PAR1-mediated and PAR2-mediated regulation of glutamatergic and GABAergic transmission, supports that PARs are important modulators of synaptic efficacy and plasticity in normal conditions.

## 1. Protease-Activated Receptors (PARs) and Their Ligands in the Nervous System

### 1.1. PARs Activation and Signaling

Protease-activated receptors (PARs) are a family of G protein-coupled receptors (GPCRs), which includes four members (PAR1, PAR2, PAR3, and PAR4) belonging to group A rhodopsin-like GPCR subfamily. PARs have an exclusive mechanism of activation, which requires a site-specific proteolytic cleavage in their *N*-terminal extracellular domain. This exposes a tethered ligand that binds to the same receptor, activating it [1].

Prototypical PARs activators are serine proteases, firstly recognized as coagulation factors, like thrombin, tissue plasminogen activator (tPA), factor Xa (FXa), factor VIIa (FVIIa), activated protein C (APC), and plasmin. Other PARs activators are trypsins, proteases released from leukocytes, like cathepsin G, elastase, and proteinase 3, as well as cell-surface proteases as membrane-type serine protease 1 (MT-SP1), and the cysteine protease, calpain [1,2,3]. Additionally, PARs can be activated by various matrix metalloproteinases (MMPs), such as MMP-1, MMP-2, MMP-3, MMP-8, MMP-9, and MMP-13, by proteolysis at non-canonical sites [3,4]. While some proteases can activate multiple PARs, other ones specifically cleave one PAR subtype (Table 1). Actually, thrombin activates PAR1, PAR3, and PAR4, although with different potencies, but does not stimulate PAR2, which is instead cleaved by trypsin and tryptase, besides other coagulation factors. The same protease can produce opposite effects on different PARs subtypes, as in the case of cathepsin G, a neutrophil serine protease, that disarms PAR1, by cleaving it into non-functional parts, while activating PAR2 and PAR4, by proteolysis that release tethered ligands [1,2,3,4,5].

In addition to the proteolytic activation, PARs can be stimulated by short peptides corresponding with the tethered ligand sequence. These peptides are able to induce PARs stimulation in the absence of proteolytic cleavage because they replace endogenous PARs-bound ligands in the activation-binding sites. Such alternative modality of activation allows a more controlled PARs activation, and is useful for distinguishing PARs functions devoid of side-effects due to protease-dependent cleavage of additional targets. The PAR1-tethered ligand peptide is SFLLR-NH_2_. Besides PAR1, it also activates PAR2, though with a minor efficacy [6], but modification in the first amino acid leads to TFLLR-NH_2_, which is a specific PAR1 activator. The PAR2-tethered sequence is SLIGKV-NH_2_, while TFRGAP-NH_2_ is the tethered ligand for PAR3, and GYPGQV-NH_2_ for PAR4 [1] (Table 1).

**Figure 1:**
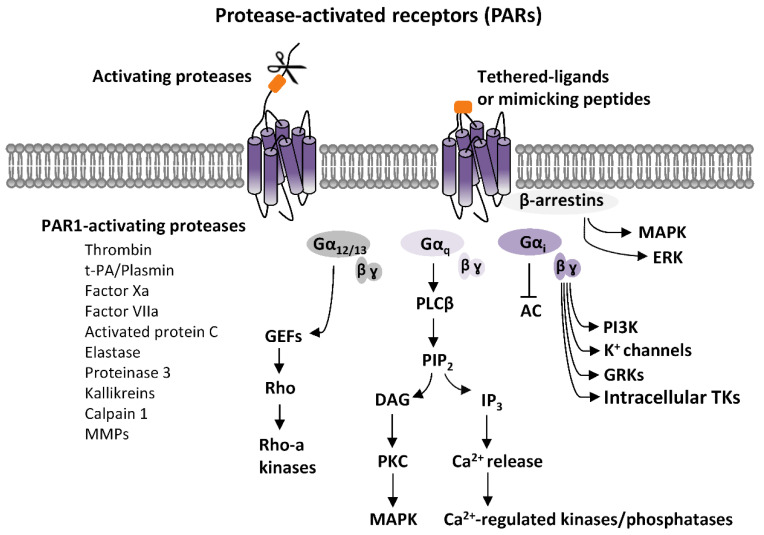
Protease-activated receptors (PARs) signaling. Scheme of the principal PARs-dependent signaling pathways. PARs activation, elicited by proteases-induced unmasking of tethered ligands or, alternatively, by ligand-mimicking peptides, stimulates various G proteins-dependent pathways. Gα_q_-mediated activation of phospholipase C β (PLCβ) results in the hydrolysis of phosphatidylinositol and generation of inositol-1,4,5-trisphosphate (IP_3_) and diacylglycerol (DAG), that fosters Ca^2+^ intracellular mobilization from internal stores, and activation of Ca^2+^-regulated kinases and phosphatase. Protein kinase C (PKC), which is also activated by DAG, stimulates mitogen-activated protein kinases (MAPK). PARs coupling with Gα_12/13_, that binds to guanine nucleotide exchange factors (GEFs), results in the activation of the small soluble G protein, Rho, and, consequently, of Rho-activated kinases. PARs-induced activation of Gi, inhibits, via Gαi,, adenylyl cyclase (AC), and, via the βγ subunit, produces opening of G protein-activated inward rectifying K^+^ channels (GIRK), the activation of G protein-coupled receptor kinases (GRKs), as well as the stimulation of intracellular tyrosine kinases (TKs), and the activation of phosphotydil-inositole-3-kinase (PI3K), that then activates other kinases signaling pathways, including mitogen-activated protein kinase (MAPK). Additionally, PARs stimulations can activate G protein-independent mechanisms, mediated by the recruitment of β-arrestins and activation of diverse signaling pathways, including MAPK-like extracellular signal-regulated kinases (ERK).

PARs activation elicits an intricate network of intracellular signaling pathways. PARs are coupled to various G proteins—G_q_, G_i_, and G_12/13_—and, additionally, they can also activate G protein-independent signaling mechanisms [1,2,3,4,5,6,7]. More information is available on PAR1, which is the first member of PARs’ family to be identified. Canonical PAR1 activation, consequent to cleavage-induced exposition of SFLLR-NH_2_ sequence, results in multiple G protein-dependent and G protein-independent signaling pathways (Figure 1). Through Gα_q_, PAR1 activates phospholipase C β (PLCβ), thereby triggering phosphoinositide hydrolysis, with the generation of inositol-1,4,5-trisphosphate (IP_3_) and diacyl-glycerol (DAG), thus, leading to Ca^2+^ mobilization from intracellular stores and activation of protein kinase C (PKC). This results in the activation of various Ca^2+^-regulated kinases and phosphatase. PAR1 coupling with Gα_12/13_, that binds to guanine nucleotide exchange factors (GEFs), results in the activation of the small soluble G protein, Rho, and consequently of Rho-activated kinases. Furthermore, PARs activation, by coupling with G_i/o_, induces the inhibition of adenylyl cyclase (AC), whereas via the βγ subunit, induces the opening of K^+^ channels (namely G protein-activated inward rectifying K^+^ channels, GIRK), the activation of G protein-coupled receptor kinases (GRKs), as well as the stimulation of non-receptor tyrosine kinases, and the activation of phosphotydil-inositole-3-kinase (PI3K), that induces the activation of other kinases signaling pathways, including mitogen-activated protein kinase (MAPK) [1,2,3,4,7] (Figure 1).

G-protein independent mechanisms, associated to PARs stimulation, are reliant on two multifunctional adaptor and transducer molecules, β-arrestins 1 and 2, that direct the recruitment, scaffolding, and activation of cytoplasmic signaling complexes, thus, activating various effectors, including MAPK and PI3K, along with being involved in the regulation of PARs internalization [1,2,3,4,7].

PAR2 activation, by trypsin that unmasks the tethered ligand SLIGKV (human) or SLIGRL (mouse), results in the stimulation of Gα_q_-, Gα_12/13_-, G_i_-associated pathways, inducing Ca^2+^ mobilization, reduced cAMP production, and activation of Rho kinases [1,2,3,4,5,6,7]. Additional G protein-independent mechanisms, downstream to PAR2 stimulation, similarly to PAR1, include recruitment of β-arrestin 1/2 and ERK activation [7].

PAR3 cleavage, by thrombin, reveals the tethered-ligand sequence TFRGAP sequence. Divergent by the other PARs subtypes, PAR3 seems not to be activated by peptides mimicking the tethered ligand sequence. Thus, it is considered able to signal only in the presence of other PARs subtypes, as obligatory dimers [8]. Though, some in vitro evidence on thrombin-induced PAR3-mediated intracellular mechanisms have been reported [9]. Canonical PAR4 activation, by thrombin and trypsin, is elicited by unmasking the tethered ligand domain GYPGQV, and mainly induces activation of G_q_-dependent and G_12/13_-dependent signaling mechanisms [7].

PARs display biased agonism, which is the phenomenon by which various ligands promote different signaling responses while activating the same receptors [3,10]. For PAR1, it has been reported that proteases acting at conventional sites, like thrombin, exposing the SFLLR-NH_2_ sequence, activate preferentially Gα_q_-dependent and Gα_12/13_-dependent pathways, whereas other proteases, like elastase and proteinase-3, uncovering a longer sequence of the tethered ligand, mainly induce Gα_i_-dependent signaling pathways, regardless of their distinct cleavage positions. Moreover, MMPs, which cleave PAR1 at non-canonical sites, that mainly cause PRSFLLR-NH_2_ release, favorably stimulate Gα_12/13_-dependent signaling pathways, producing activation of MAPK [3,10].

PARs can form homodimers or heterodimers, and dimerization possibly causes activation of other signaling pathways additional to monomers-activated ones [3,4]. Moreover, a tethered ligand of an active PAR can trans-activate an uncleaved PAR. This has been demonstrated for the PAR1-tethered ligand, which can activate PAR2, while this crosstalk is not bidirectional, since the PAR2-tethered ligand cannot stimulate PAR1 [1]. Such crosstalk, along with PARs dimerization, possibly boosts protease-mediated effects. In the PAR1-PAR2 dimer, thrombin-induced cleavage of PAR1, leads to exposure of tethered-ligand that can transactivate PAR2 (which is not a canonical thrombin substrate), thus, allowing thrombin to trigger PAR2-dependent signaling pathways.

Beyond their own signaling, PARs can transactivate various receptors, either GPCR or receptors tyrosine kinases receptors (RTKs) [2]. Such PAR1-mediated transactivation can be reliant on the rapid release of agonists (like in the case of prostaglandins, and epidermal growth factor receptor (EGFR)) or be induced by stimulation of intracellular mediators, then targeting and activating the second receptor. An additional modality of transactivation, is the interaction—direct or indirect—between PARs and signal mediators downstream to GPCR, that can occur through direct physical interaction [2]. PARs crosstalk with a plethora of other receptors and signal transducers, like various GPCRs and ion channels, as well as receptor tyrosine kinases (RTKs), and receptor serine-threonine kinases (RSTKs). Among GPCRs, PARs’ interplay is described for the angiotensin receptor AT-1, serotoninergic 5-HT_2A_, purinergic P2Y12, sphingosine-1-phosphate receptor 1 (S1PR1), bradykinin B2 receptor (B2R), and prostaglandin E receptor (EP), whereas PARs can functionally interact with RTKs activated by several neurotrophic factors, like EGFR, insulin growth factor receptor (IGF-1R), fibroblast growth factor receptor (FGFR), platelet-derived growth factor receptor (PDGFR), vascular epidermal growth factor receptor (VEGFR), or RSTKs, such as ALK1-ALK7, toll-like receptors (TLRs) as TLR3 and TLR4, NOD-like receptors, and Cargo receptors.

Additionally, a functional interplay has been reported between PARs and different ligand-gated ion channels, including glutamatergic NMDARs, purinergic P2X1, and members of the family of transient receptor potentials channels, namely TRPA1, TRPV1, and TRPV4 [2]. Overall, such intricate crosstalk greatly expands the cellular effects attributable to PARs activation, contributing to ultimately determine factual PARs functions.

### 1.2. PARs Activation in the Brain

PARs have well-recognized roles in coagulation, hemostasis, and inflammation, and have been deeply investigated for their function in cellular survival/degeneration processes [1]. In addition to effects in peripheral systems, it is becoming overt that PARs have important roles in the central nervous system (CNS). Research of such functions has been alimented by early detections of cerebral expression of different PARs subtypes [11,12,13,14,15,16]. PARs’ family includes four members (PAR1, PAR2, PAR3, and PAR4). PAR1, which is the first to be identified, has been originally termed as‘thrombin receptor’ [17]. Beyond its initial identification in platelets, PAR1 expression has been reported in several organs and different cell types, including endothelial cells, fibroblasts, monocytes, T-cell lines, smooth muscle cells, and in organs such as stomach, colon, kidney, testis, eye, and brain [1]. In the brain, PAR1 is ubiquitously expressed, being found in the prefrontal cortex, basal ganglia, dorsal striatum, nucleus accumbens, substantia nigra, ventral tegmental area, amygdala, and hippocampus. Its localization has been reported either in neurons, or in astrocytes and microglia, even though there are strong differences among different brain areas and cellular populations [11,13,14,18,19,20].

PAR2 has been identified following PAR1 as a receptor for the serine protease trypsin [21]. PAR2 expression has been reported in both the human and rodent CNS in various areas, including the hippocampus (through CA1, CA2, and CA3 areas and the granular cell layer of the dentate gyrus), as well as cortex, amygdala, thalamus, hypothalamus, substantia nigra, and striatum [12,14,22,23].

PAR3, earlier described as a second thrombin receptor [24], displays a similar cerebral expression of PAR2, being localized in various hippocampal and cortical areas, as well as in amygdala, thalamus, hypothalamus, and striatum [14].

Brain localization of PAR4, firstly known as a receptor for both thrombin and trypsin, has been described in the hippocampus and cortex, thalamus, hypothalamus, and amygdala [14]. While it is documented that all PARs display a broad expression in the various brain areas, and some indication on regions of highest expression have been revealed, a deeper analysis to expose overlapping vs. segregate expression of distinct subtypes in sub-regions or cellular populations remains to be completed.

Evidence of brain expression of PARs has been complemented with the demonstration of resident sources of PARs-activating proteases in the brain. Such data has challenged the earlier belief about PARs activation in the brain occurring only in pathological conditions allowing influx of peripheral proteases, through an impaired blood-brain barrier (BBB), and it is now accepted that PARs-activating proteases can be released from neurons, astrocytes, microglia, or other immunity cells that are resident (or recruited) in the brain, in addition to being derived from the circulation [1,11,17].

Specifically, there is evidence documenting brain synthesis of the prototypical PARs activator, thrombin, with both a mature or precursor form, pro-thrombin, found in several brain areas [11,16,18,25,26,27]. Likewise, tPA can be released by neurons, glial cells, and endothelial cells, being highly expressed in various brain regions, including the cerebellum, cortex, amygdala, and hippocampus [27,28,29,30,31,32]. Further evidence supports brain expression of other PARs-activating proteases, including trypsin [33], and trypsin-like proteases, such as MSP and kallikreins [34,35], besides MMPs [36].

Actions of cerebral PARs-activating proteases is tightly regulated. Serine proteases activity in CNS is tempered by another class of proteins, i.e., the serine protease inhibitors (SERPINs), including protease nexin-1 (PN-1), neuroserpin, and antithrombin 3 (AT3) [27,37,38,39]. The activity of such SERPINs, by influencing PARs-activating proteases, can indirectly affect PARs signaling/function in the brain.

Hence, it is currently established that, in a normal brain, there are necessary elements—PARs activators and receptors—to permit physiological PARs signaling. Beyond such a physiological tone, levels of PARs-activating proteases possibly boost during some conditions, like inflammation or trauma, that either recruit additional proteases-releasing cells types, or increase BBB permeability, fostering coagulation cascade proteases inflow in the CNS from the periphery [1], with the consequence of an abnormal PARs activation. Actually, multifaceted PARs roles have been previously reported in neuroinflammatory and neurodegenerative processes in diverse cerebral illnesses, in stroke, brain trauma, Alzheimer’s disease (AD), and Parkinson’s disease (PD). Likewise, aberrant activity of serine proteases and MMPs, possibly resulting in abnormal PARs signaling, has been linked to AD, PD, TBI, stroke, epilepsy, and familial encephalopathy with neuroserpin inclusion bodies (FENIB) [3,5,36,40,41,42,43,44,45].

Differently from their pathological relevance, physiological roles for PARs in the brain have been less appreciated. Nevertheless, it is becoming clear that PARs have a “neuromodulatory” function, affecting neurotransmission and synaptic plasticity in a normal brain, thus, possibly contributing to either learning and memory processes and complex behaviors.

## 2. PARs’ Roles in the Regulation of Neurotransmission and Synaptic Plasticity

It is well-appreciated that serine proteases and MMPs, as well as their zymogen precursors and endogenous inhibitors, are modulators of synaptic functions in various brain areas, including hippocampus, striatum, and cortex, thus, affecting synaptic plasticity, as well as learning and memory processes and behaviors. Pioneering investigations have revealed a role for tPA in the regulation of basal glutamatergic transmission and forms of long-term potentiation (LTP) in the hippocampus [46,47,48,49] and in the striatum [50]. Such evidence about tPA-mediated facilitation of glutamatergic synaptic plasticity has been later supplemented by description of effects of other serine proteases, including thrombin [51,52], on glutamatergic synaptic plasticity, as well as the recognition of serine proteases’ contribution, in parallel to synaptic changes, for learning and memory processes and behaviors [49,50,53,54,55].

Although mechanisms by which serine proteases affect synaptic transmission might be multiple—i.e., including the direct proteolysis of extracellular matrix, activation/inhibition of other proteases, or direct cleavage of synaptic molecules that finalize changes in synaptic efficacy—PARs can be their direct and primary targets, and then activating intracellular cascade events that foster enduring modifications of synaptic strength. Actually, specific evidence for a direct involvement of PARs in the regulation of neurotransmission and synaptic plasticity have been provided by analyzing the functional effects of pharmacological PARs modulation, and their genetic deletion. Available evidence is focused on PAR1-mediated and PAR2-mediated effects, and mainly restricted to glutamatergic transmission, with fewer information on GABAergic transmission.

### 2.1. PARs-Dependent Modulation of Glutamatergic Transmission

The first evidence about PARs-dependent regulation of glutamatergic transmission has been reported in the hippocampus, with the demonstration that PAR1 potentiates NMDARs functions in pyramidal neurons of the CA1 area [56,57,58]. Such an increase in NMDARs-activated currents in CA1 pyramidal neurons from hippocampal rodent slices is observed after PAR1 activation with either proteases, like thrombin and plasmin, or tethered ligand-mimicking peptides, and occurs through a mechanism prompted by PAR1 stimulation on astrocytes [56,57,58]. This PAR1-dependent enhancement of glutamatergic transmission in hippocampal CA1 pyramidal neurons appeared selective for NMDARs, while AMPARs function was not affected [58]. Insights on cellular mechanisms by which PAR1 stimulation results in the potentiation of glutamatergic transmission in hippocampal CA1 pyramidal neurons depict a scenario in which PAR1 activation on astrocytes, by increasing Ca^2+^ levels in astrocytic microdomains, leads to astrocyte-released glutamate, through Ca^2+^-activated anion channels, namely Bestrophin-1 (Best-1) channels [59], with PAR1-mediated potentiation of glutamatergic transmission being ultimately mediated by GluN2A-containing NMDARs on CA1 pyramidal neurons [59]. In line with a facilitation of NMDARs-dependent responses, PAR1 stimulation produces a long-term potentiation (LTP) of field excitatory postsynaptic potentials (fEPSPs) at Schaffer collaterals-CA1 synapses in rat hippocampal slices, increasing the NMDAR-mediated component [59,60]. Accordingly, PAR1 genetic deletion affects NMDAR-dependent synaptic plasticity in the hippocampus, impairing theta burst stimulation (TBS)-induced LTP of fEPSPs at Schaffer collaterals-CA1 synapses. This evidence further corroborates the key function of PAR1 in long-term regulation of glutamatergic synaptic transmission in the hippocampus [61].

**Figure 2:**
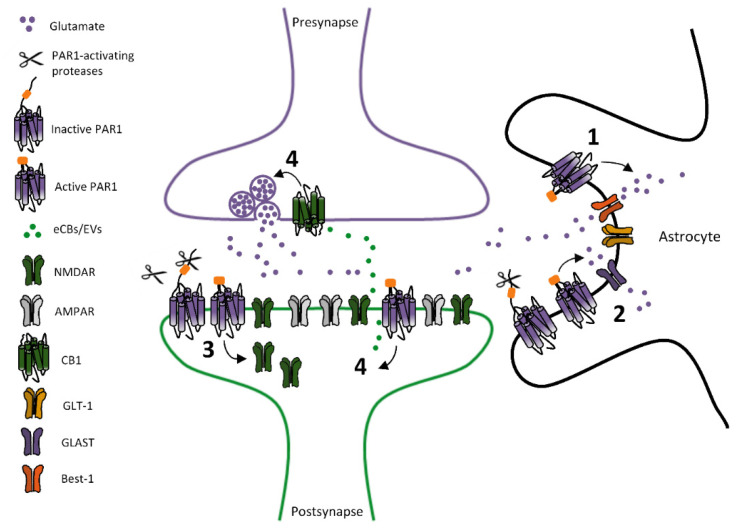
PAR1-dependent modulation of glutamatergic transmission. Scheme depicting proposed mechanisms underlying PAR1-induced regulation of glutamatergic transmission. They include (1) PAR1 activation fostering glutamate release from astrocytes and diffusion through Bestrophin-1 (Best-1) channels. (2) Astrocytic PAR1 activation modulating glutamate reuptake by glutamate transporter 1 (GLT-1) or glutamate aspartate transporter (GLAST). (3) PAR1-dependent endocytosis of NMDARs. (4) PAR1-induced endocannabinoids (eCBs)/endovanilloids (EVs)-dependent reduction of glutamate release.

Additional PAR1’s modulatory roles have been later revealed, supporting multifaceted effects of PAR1 stimulation—either causing potentiation or weakening of glutamatergic transmission—through multiple mechanisms occurring at both postsynaptic or presynaptic neuronal loci, or in glial cells (Figure 2). Particularly, a bidirectional PAR1-mediated control of glutamate transmission discloses a brain area/cellular population-linked heterogeneity of mechanisms downstream to PAR1 stimulation. Actually, besides potentiating NMDARs function, PAR1 stimulation in hippocampal CA1 area has been associated with a reduction of glutamatergic transmission. In particular, PAR1 activation decreases AMPARs-mediated and NMDAR-mediated component of glutamatergic transmission in CA1 pyramidal neurons from hippocampal mouse slices, by an astrocyte-mediated regulation of synaptic glutamate levels [62]. Accordingly to this evidence, PAR1 stimulation, by fostering glutamate clearance by astrocytes, weakens glutamatergic basal transmission synaptic plasticity, affecting LTP of fEPSPs at Schaffer collaterals-CA1 synapses [62].

Beyond the hippocampus, PAR1-dependent modulation of glutamatergic transmission has been reported in other areas, both in the central and peripheral nervous system. In the caudal nucleus of the solitary tract (NST), astrocytic PAR1 stimulation potentiates basal glutamatergic transmission on adjacent neurons in rat brain slices, by regulating glutamate release that potentiates NMDARs-mediated currents, favorably acting on the GluN2A-containing NMDARs pool [63]. PAR1-mediated potentiation of basal glutamatergic transmission in rat NST neurons has been reported in another study [64], which describes that PAR1 activation on astrocytes fosters endovanilloids (EVs) release and EVs-induced activation of transient receptor potential vanilloid 1 (TRPV1) on glutamatergic presynaptic terminals, thus, regulating glutamate release [64]. Likewise, PAR1 modulates glutamatergic transmission in substantia gelatinosa of the spinal cord (Layer II neurons), where PAR1 activation increases spontaneous glutamatergic transmission through a presynaptic modulation of glutamate release, as demonstrated by increased frequency of spontaneous excitatory postsynaptic currents in neurons from rat spinal cord slices [65].

PAR1-dependent regulation of glutamatergic transmission has been recently described in mouse midbrain dopamine (DA) neurons of substantia nigra pars compacta (SNpc) [66]. Herein, PAR1 stimulation induces a hypofunction of NMDARs—namely a long-term depression (LTD) of NMDAR-mediated synaptic currents—reliant on a PAR1-induced endocytosis of NMDARs in nigral DA neurons [66]. Precisely, synaptic GluN2B/GluN2D-containing NMDARs are inhibited following PAR1 stimulation in SNpc slices, whereas, synaptic AMPARs are not affected, thus, supporting that PAR1’s modulatory roles on fast glutamatergic transmission in SNpc DA neurons are focused on the NMDAR-mediated component. Of note, PAR1 inhibition slightly limits the downregulation of NMDARs-mediated responses, observed during repetitive stimulations, and this supports that PAR1 tonically controls surface levels of NMDARs on DA neurons [66].

Evidence on PARs crosstalk with metabotropic glutamate receptors (mGluRs) are limited to the observation that PAR1 activation, with thrombin and/or a ligand-mimicking peptide, downregulates the expression of the mGluR5 mRNA and protein in cultured astrocytes [67]. Implications of such PAR1-induced regulation of astrocytic mGluR5 have not been further elucidated. In addition, it is lacking any evaluation of PAR1-dependent regulation of mGluR5 (and other mGluRs) expression in further cellular populations other than astrocytes.

Emerging evidence also supports that PAR2, in addition to PAR1, can affect glutamatergic transmission and plasticity [68,69]. PAR2 activation induces an LTD of glutamatergic transmission in rat hippocampal CA1 area in acute slices through an NMDAR-dependent mechanism, which is mediated by GluN2B-containing receptors [68]. mGluRs, that are usually chief players in the induction of glutamatergic LTD at hippocampal CA3-CA1 synapses, are not involved in this PAR2-induced form of glutamatergic synaptic plasticity [68]. As the underlying mechanism, a PAR2-dependent modulation of astrocytes-released glutamate has been proposed, but more evidence needs to clarify mechanistic events that lead to NMDAR-dependent LTD induction, following PAR2 activation. PAR2-induced glutamatergic LTD at hippocampal CA3-CA1 synapses has been confirmed in another study in mouse brain slices, wherein it has been reported that PAR2-induced LTD is reliant on an interplay between NMDARs and transient receptor potential vanilloid 4 (TRPV4), also involving the activation of protein kinase A (PKA) [69].

In conclusion, available evidence points to an important role of PARs in the modulation of glutamatergic transmission (Table 2). PARs can either potentiate or inhibit glutamatergic transmission, by means of multiple mechanisms, which can be either cell-autonomous or involve the interplay of other cellular populations (neurons and/or astrocytes). PARs localization—segregated in distinct brain areas, or diverse cellular types in the same compartment—appears as a main determinant of engaged mechanisms. In the hippocampus, where PAR1 is especially located on astrocytes, PAR1-dependent mechanisms on glutamatergic transmission in neurons are more indirect (mainly due to astrocyte-dependent regulation of glutamate synaptic levels), while, in other brain areas, where PAR1 expression is preferentially neuronal, changes in glutamatergic transmission are directly reliant on cell-autonomous mechanisms or due to retrogradely acting neuron-released mediators.

NMDARs represent preferential PARs targets, though area-specific differences have been reported sustaining either potentiation or inhibition of NMDARs function, by activation of a diverse PARs subtype (PAR1 and PAR2). AMPARs function seems to be less modulated by PARs stimulation, whereas crosstalk between PARs and mGluRs is still largely unexplored.

Among functional outcomes of PARs-mediated regulation of glutamate receptors are long-lasting modifications in synaptic plasticity. In the hippocampus, PAR1- and PAR2 seem to have opposite effects on glutamatergic synaptic plasticity (with PAR1 fostering LTP and PAR2 inducing LTD), thus, differently contributing in the LTP/LTD balance, that ultimately define synaptic strength in the hippocampal circuitry [52]. In midbrain DA neurons, otherwise, PAR1 activation induces LTD of NMDAR-mediated transmission [66], thus, supporting that brain areas-related differences exist in the role each PAR subtype has in controlling glutamatergic synaptic plasticity.

Altogether, current evidence points to a key role for PARs in regulating glutamatergic transmission, and, certainly, performing similar functional analyses in other brain areas/neuronal populations will expand the factual PARs relevance on the control of glutamatergic synaptic transmission and plasticity.

### 2.2. PARs-Dependent Modulation of GABAergic Transmission

Evidence on PARs-dependent modulation of GABAergic transmission are limited to a few studies. It has been reported that PAR1 activation inhibits evoked GABAergic transmission in hippocampal neuronal cultures, by fostering the synthesis of endocannabinoids (eCB), that retrogradely activate cannabinoids receptor 1 (CB1) on presynaptic GABAergic terminals, and, thus, reduce GABA release [70]. Additional analyses performed in brain slices might confirm if such PAR1-induced eCB-mediated control of GABA release is also relevant to regulate GABAergic transmission in more intact physiological tissues. Another PAR2-dependent effect on GABAergic transmission has been described in sensory neurons in spinal cord dorsal horn slices, wherein a PAR2-activating peptide reduces spontaneous GABAergic transmission, by mixed presynaptic or postsynaptic mechanisms, as demonstrated by reduced frequency and amplitudes of GABAergic spontaneous inhibitory postsynaptic currents (sIPSCs) [71].

Overall, available evidence mainly suggests an inhibitory role of PARs in regulating spontaneous and evoked GABAergic transmission (Table 2). However, further investigations warrant to clarify the factual relevance of PARs in the control of GABAergic transmission, and to unveil if they can produce enduring effects on inhibitory synaptic plasticity.

## 3. Conclusions and Perspectives

Currently, in spite of a documented brain expression of PARs and of their activating proteases, cerebral PARs’ roles are still barely appreciated. Nevertheless, the growing evidence, supporting PARs’ functions in the regulation of neurotransmission and synaptic plasticity, identifies PARs as important mediators controlling key functions of a normal brain. Such evidence further enlarges the functional relevance of proteases—far to be just considered mere destructive/digestive enzymes and now well-recognized “neuromodulators”—that, by activation of diverse PARs subtypes, can finely and bidirectionally adjust synaptic functioning and neuronal communication.

The importance of PARs in affecting neuronal communication so far identified should encourage in the research for novel PARs-dependent functions in diverse brain areas/cellular populations. Going through this unexplored field might lead to discover novel mechanisms that govern brain functioning either in health or in diseases.

## Figures and Tables

**Table 1:** Protease-activated receptors (PARs): activation, brain localization, and signaling.

Receptor	Activating Proteases	Inactivating Proteases	Activating Peptides	Signaling Pathways	Cerebral Localization
PAR1	Thrombin Factor VIIa (FVIIa) Factor Xa (FXa) Plasmin MMP-1, -2, -3, -8, -9, -12, -13 Activated protein C (APC) Elastase Proteinase 3 Kallikrein 4, -5, -6, -14 Granzyme A, B, K Calpain-1	Cathepsin G Proteinase 3 Elastase Plasmin Chymase	SFLLR-NH_2_ TFLLR-NH_2_ NPNDKYEPF-NH_2_ PRSFLLR-NH_2_	G_q_ G_i_ G_12/13_ β-arrestins	Hippocampus Cortex Amydgala Substantia nigra Ventral tegmental Area Thalamus Hypothalamus Striatum Dorsal root ganglion
PAR2	Trypsin I/II Trypsin IV Tryptase Factor VIIa (FVIIa) Factor Xa (FXa) Elastase Proteinase 3 Cathepsin G Acrosin Granzyme A Kallikrein 2, -4,-5, -6, 14	Chymase	SLIGRL-NH_2_ SLIGKV-NH_2_ AC-98170 AC-55541	G_q_ G_i_ G_12/13_ β-arrestins	Hippocampus Cortex Amydgala Substantia nigra Thalamus Hypothalamus Striatum Dorsal root ganglion
PAR3	Thrombin Trypsin Activated protein C (APC)	Cathepsin G	TFRGAP-NH_2_	G_q_	Hippocampus Cortex Amydgala Thalamus Hypothalamus Striatum Dorsal root ganglion
PAR4	Thrombin Trypsin Plasmin Cathepsin G MT-SP1		GYPGQV-NH_2_ GYPGKF-NH_2_ AYPGKF	G_q_ G_12/13_	Hippocampus Cortes Amydgala Thalamus Hypothalamus

**Table 2:** Roles of PARs (PAR1 and PAR2) in the modulation of neurotransmission and synaptic plasticity.

Receptor	Neurotransmitter System	Effect	Main Response/Mechanism	Brain Area/Cellular Population	References
PAR1	Glutamatergic Transmission	↑	Increased NMDAR-mediated spontaneous EPSCs	Hippocampus CA1 area, Pyramidal neurons	[56]
		↑	Potentiated NMDA-activated currents and NMDARs-mediated spontaneous EPSCs, due to PAR1-induced glutamate release	Hippocampus CA1 Area, Pyramidal neurons	[57,58]
		↑	NMDAR-dependent LTP of field EPSPs	Hippocampus, CA3-CA1 synapses	[60]
		↑	Increased NMDAR-mediated currents and LTP of fEPSPs, due to astrocyte-released glutamate via Best-1 channels	Hippocampus, CA3-CA1 synapses	[59]
		↑	Impaired NMDAR-dependent TBS-induced LTP of fEPSPs in PAR1 knockout mice	Hippocampus, CA3-CA1 synapses	[61]
		↑	Potentiated NMDAR-mediated spontaneous transmission, due to astrocytic PAR1-induced glutamate release	Nucleus of solitary tract, neurons	[63]
		↑	Increased glutamate release, elicited by astrocytic PAR1-released endovanilloids (EVs) and TRPV1 activation	Nucleus of solitary tract, neurons	[64]
		↑	Increased spontaneous EPSCs, due to enhanced glutamate release	Spinal cord, Substantia gelatinosa neurons	[65]
		↓	Reduced NMDAR-mediated EPSCs and decreased NMDAR-dependent LTP of field EPSPs	Hippocampal CA3-CA1 synapses	[62]
		↓	Reduced AMPAR-mediated EPSCs	Hippocampal CA1 area, pyramidal neurons	[62]
		↓	LTD of NMDAR-mediated EPSCs, due to PAR1-induced NMDARs endocytosis	Substantia nigra compacta, DA neurons	[66]
		↓	Reduced NMDA-activated currents	Substantia nigra compacta, DA neurons	[66]
		=	Unaffected synaptic and extrasynaptic AMPAR-mediated currents	Substantia nigra compacta, DA neurons	[66]
		↓	Reduced mGluR5 expression	Astrocytic cultures	[67]
PAR1	GABAergic transmission	↓	Reduced spontaneous and evoked IPSCs due to PAR1-dependent eCB-mediated decrease of GABA release	Hippocampal neuronal cultures	[70]
PAR2	Glutamatergic transmission	↓	LTD of fEPSPs (NMDAR-mediated)	Hippocampus, CA3-CA1 synapses	[68]
		↓	LTD of fEPSPs (TRPV4-mediated)	Hippocampus, CA3-CA1 synapses	[69]
	GABAergic transmission	↓	Reduced spontaneous IPSCs	Spinal cord dorsal horn, neurons	[71]

## References

[1] Ossovskaya V.S., Bunnett N.W. (2004). Protease-activated receptors: Contribution to physiology and disease. Physiol. Rev..

[2] Gieseler F., Ungefroren H., Settmacher U., Hollenberg M.D., Kaufmann R. (2013). Proteinase-activated receptors (PARs)—Focus on receptor-receptor-interactions and their physiological and pathophysiological impact. Cell Commun. Signal..

[3] Heuberger D.M., Schuepbach R.A. (2019). Protease-activated receptors (PARs): Mechanisms of action and potential therapeutic modulators in PAR-driven inflammatory diseases. J. Thromb..

[4] Fox O.W., Preston R.J.S. (2020). Molecular basis of protease-activated receptor 1 signaling diversity. J. Thromb. Haemost..

[5] Ramachandran R., Noorbakhsh F., DeFea K., Hollenberg M.D. (2012). Targeting proteinase-activated receptors: Therapeutic potential and challenges. Nat. Rev. Drug Discov..

[6] Blackhart B.D., Emilsson K., Nguyen D., Teng W., Martelli A.J., Nystedt S., Sundelin J., Scarborough R.M. (1996). Ligand cross-reactivity within the protease-activated receptor family. J. Biol. Chem..

[7] Soh U.J., Dores M.R., Chen B., Trejo J. (2010). Signal transduction by protease-activated receptors. Br. J. Pharm..

[8] Nakanishi-Matsui M., Zheng Y.W., Sulciner D.J., Weiss E.J., Ludeman M.J., Coughlin S.R. (2000). PAR3 is a cofactor for PAR4 activation by thrombin. Nature.

[9] Seminario-Vidal L., Kreda S., Jones L., O’Neal W., Trejo J., Boucher R.C., Lazarowski E.R. (2009). Thrombin promotes release of ATP from lung epithelial cells through coordinated activation of rho- and Ca^2+^-dependent signaling pathways. J. Biol Chem..

[10] Zhao P., Metcalf M., Bunnett N.W. (2014). Biased signaling of protease-activated receptors. Front. Endocrinol..

[11] Weinstein J.R., Gold S.J., Cunningham D.D., Gall C.M. (1995). Cellular localization of thrombin receptor mRNA in rat brain: Expression by mesencephalic dopaminergic neurons and codistribution with prothrombin mRNA. J. Neurosci..

[12] D’Andrea M.R., Derian C.K., Leturcq D., Baker S.M., Brunmark A., Ling P., Darrow A.L., Santulli R.J., Brass L.F., Andrade-Gordon P. (1998). Characterization of protease-activated receptor-2 immunoreactivity in normal human tissues. J. Histochem. Cytochem..

[13] Niclou S.P., Suidan H.S., Pavlik A., Vejsada R., Monard D. (1998). Changes in the expression of protease-activated receptor 1 and protease nexin-1 mRNA during rat nervous system development and after nerve lesion. Eur. J. Neurosci..

[14] Striggow F., Riek-Burchardt M., Kiesel A., Schmidt W., Henrich-Noack P., Breder J., Krug M., Reymann K.G., Reiser G. (2001). Four different types of protease-activated receptors are widely expressed in the brain and are up-regulated in hippocampus by severe ischemia. Eur. J. Neurosci..

[15] Wang H., Ubl J.J., Reiser G. (2002). Four subtypes of protease-activated receptors, co-expressed in rat astrocytes, evoke different physiological signaling. Glia.

[16] Sokolova E., Reiser G. (2008). Prothrombin/thrombin and the thrombin receptors PAR-1 and PAR-4 in the brain: Localization, expression and participation in neurodegenerative diseases. Thromb. Haemost..

[17] Vu T.K., Hung D.T., Wheaton V.I., Coughlin S.R. (1991). Molecular cloning of a functional thrombin receptor reveals a novel proteolytic mechanism of receptor activation. Cell.

[18] Dihanich M., Kaser M., Reinhard E., Cunningham D., Monard D. (1991). Prothrombin mRNA is expressed by cells of the nervous system. Neuron.

[19] Balcaitis S., Xie Y., Weinstein J.R., Andersen H., Hanisch U., Ransom B.R., Möller T. (2003). Expression of proteinase-activated receptors in mouse microglial cells. Neuroreport.

[20] Junge C.E., Lee C.J., Hubbard K.B., Zhang Z., Olson J.J., Hepler J.R., Brat D.J., Traynelis S.F. (2004). Protease-activated receptor-1 in human brain: Localization and functional expression in astrocytes. Exp. Neurol..

[21] Nystedt S., Emilsson K., Wahlestedt C., Sundelin J. (1994). Molecular cloning of a potential proteinase activated receptor. Proc. Natl. Acad. Sci. USA.

[22] Bushell T.J., Plevin R., Cobb S., Irving A.J. (2006). Characterization of proteinase-activated receptor 2 signalling and expression in rat hippocampal neurons and astrocytes. Neuropharmacology.

[23] Lohman R.J., O’Brien T.J., Cocks T.M. (2008). Protease-activated receptor-2 regulates trypsin expression in the brain and protects against seizures and epileptogenesis. Neurobiol. Dis..

[24] Ishihara H., Connolly A.J., Zeng D., Kahn M.L., Zheng Y.W., Timmons C., Tram T., Coughlin S.R. (1997). Protease-activated receptor 3 is a second thrombin receptor in humans. Nature.

[25] Turgeon V.L., Houenou L.J. (1997). The role of thrombin-like (serine) proteases in the development, plasticity and pathology of the nervous system. Brain Res. Brain Res. Rev..

[26] Turgeon V.L., Salman N., Houenou L.J. (2000). Thrombin: A neuronal cell modulator. Thromb Res..

[27] Almonte A.G., Sweatt J.D. (2011). Serine proteases, serine protease inhibitors, and protease-activated receptors: Roles in synaptic function and behavior. Brain Res..

[28] Qian Z., Gilbert M.E., Colicos M.A., Kandel E.R., Kuhl D. (1993). Tissue plasminogen activator is induced as an immediate-early gene during seizure, kindling, and long-term potentiation. Nature.

[29] Sallés F.J., Strickland S. (2002). Localization and regulation of the tissue plasminogen activator-plasmin system in the hippocampus. J. Neurosci..

[30] Shin C.Y., Kundel M., Wells D.G. (2004). Rapid, activity-induced increase in tissue plasminogen activator is mediated by metabotropic glutamate receptor-dependent mRNA translation. J. Neurosci..

[31] Yepes M., Lawrence D.A. (2004). New functions for an old enzyme: Nonhemostatic roles for tissue-type plasminogen activator in the central nervous system. Exp. Biol. Med..

[32] Lochner J.E., Honigman L.S., Grant W.F., Gessford S.K., Hansen A.B., Silverman M.A., Scalettar B.A. (2006). Activity-dependent release of tissue plasminogen activator from the dendritic spines of hippocampal neurons revealed by live-cell imaging. J. Neurobiol..

[33] Gschwend T.P., Krueger S.R., Kozlov S.V., Wolfer D.P., Sonderegger P. (1997). Neurotrypsin, a novel multidomain serine protease expressed in the nervous system. Mol. Cell Neurosci..

[34] Scarisbrick I.A., Isackson P.J., Ciric B., Windebank A.J., Rodriguez M. (2001). MSP, a trypsin-like serine protease, is abundantly expressed in the human nervous system. J. Comp. Neurol..

[35] Bernett M.J., Blaber S.I., Scarisbrick I.A., Dhanarajan P., Thompson S.M., Blaber M. (2002). Crystal structure and biochemical characterization of human kallikrein 6 reveals that a trypsin-like kallikrein is expressed in the central nervous system. J. Biol. Chem..

[36] Brkic M., Balusu S., Libert C., Vandenbroucke R.E. (2015). MMPs in brain disease friends or foes: Matrix metalloproteinases and their multifaceted roles in neurodegenerative diseases. Mediat. Inflam..

[37] Wagner S.L., Van Nostrand W.E., Lau A.L., Farrow J.S., Suzuki M., Bartus R.T., Schuppek R., Nguyen A., Cotman C.W., Cunningham D.D. (1993). Co-distribution of protease nexin-1 and protease nexin-2 in brains of non-human primates. Brain Res..

[38] Reinhard E., Suidan H.S., Pavlik A., Monard D. (1994). Glia-derived nexin/protease nexin-1 is expressed by a subset of neurons in the rat brain. J. Neurosci Res..

[39] Miranda E., Lomas D.A. (2006). Neuroserpin: A serpin to think about. Cell Mol. Life Sci..

[40] Junge C.E., Sugawara T., Mannaioni G., Alagarsamy S., Conn P.J., Brat D.J., Chan P.H., Traynelis S.F. (2003). The contribution of protease-activated receptor 1 to neuronal damage caused by transient focal cerebral ischemia. Proc. Natl. Acad. Sci. USA.

[41] Nicole O., Goldshmidt A., Hamill C.E., Sorensen S.D., Sastre A., Lyuboslavsky P., Helper J.R., McKeon R.J., Traynelis S.F. (2005). Activation of protease-activated receptor-1 triggers astrogliosis after brain injury. J. Neurosci..

[42] Luo W., Wang Y., Reiser G. (2007). Protease-activated receptors in the brain: Receptor expression, activation, and functions in neurodegeneration and neuroprotection. Brain Res. Rev..

[43] Davis R.L., Shrimpton A.E., Holohan P.D., Bradshaw C., Feiglink D., Collins G.H., Sonderegger P., Kinter J., Becker L.M., Lacbawan F. (1999). Familial dementia caused by polymerization of mutant Neuroserpin. Nature.

[44] Molinari F., Meskanaite V., Munnich A., Sonderegger P., Colleaux L. (2003). Extracellular proteases and their inhibitors in genetic diseases of the central nervous system. Hum. Mol. Genet..

[45] Fabbro S., Seeds N.W. (2009). Plasminogen activator activity is inhibited while neuroserpin is up-regulated in the Alzheimer disease brain. J. Neurochem..

[46] Frey U., Müller M., Kuhl D. (1996). A different form of long-lasting potentiation revealed in tissue plasminogen activator mutant mice. J. Neurosci..

[47] Lüthi A., Van Der Putten H., Botteri F.M., Mansuy I.M., Meins M., Frey U., Sansig G., Portet C., Schmutz M., Schröder M. (1997). Endogenous serine protease inhibitor modulates epileptic activity and hippocampal long-term potentiation. J. Neurosci..

[48] Baranes D., Lederfein D., Huang Y.Y., Chen M., Bailey C.H., Kandel E.R. (1998). Tissue plasminogen activator contributes to the late phase of LTP and to synaptic growth in the hippocampal mossy fiber pathway. Neuron.

[49] Madani R., Hulo S., Toni N., Madani H., Steimer T., Muller D., Vassalli J. (1999). Enhanced hippocampal long-term potentiation and learning by increased neuronal expression of tissue-type plasminogen activator in transgenic mice. EMBO J..

[50] Calabresi P., Napolitano M., Centonze D., Marfia G.A., Gubellini P., Teule M.A., Berretta N., Bernardi G., Frati L., Tolu M. (2000). Tissue plasminogen activator controls multiple forms of synaptic plasticity and memory. Eur. J. Neurosci..

[51] Maggio N., Itsekson Z., Dominissini D., Blatt I., Amariglio N., Rechavi G., Tanne D., Chapman J. (2013). Thrombin regulation of synaptic plasticity: Implications for physiology and pathology. Exp. Neurol..

[52] Ben Shimon M., Lenz M., Ikenberg B., Becker D., Shavit Stein E., Chapman J., Tanne D., Pick C.G., Blatt I., Neufeld M. (2015). Thrombin regulation of synaptic transmission and plasticity: Implications for health and disease. Front. Cell Neurosci..

[53] Pawlak R., Magarinos A.M., Melchor J., McEwen B., Strickland S. (2003). Tissue plasminogen activator in the amygdala is critical for stress-induced anxiety-like behavior. Nat. Neurosci..

[54] Pawlak R., Rao B.S., Melchor J.P., Chattarji S., McEwen B., Strickland S. (2005). Tissue plasminogen activator and plasminogen mediate stress-induced decline of neuronal and cognitive functions in the mouse hippocampus. Proc. Natl. Acad. Sci. USA.

[55] Matys T., Pawlak R., Matys E., Pavlides C., McEwen B.S., Strickland S. (2004). Tissue plasminogen activator promotes the effects of corticotropin-releasing factor on the amygdala and anxiety-like behavior. Proc. Natl. Acad. Sci. USA.

[56] Gingrich M.B., Junge C.E., Lyuboslavsky P., Traynelis S.F. (2000). Potentiation of NMDA receptor function by the serine protease thrombin. J. Neurosci..

[57] Lee C.J., Mannaioni G., Yuan H., Woo D.H., Gingrich M.B., Traynelis S.F. (2007). Astrocytic control of synaptic NMDA receptors. J. Physiol..

[58] Mannaioni G., Orr A.G., Hamill C.E., Yuan H., Pedone K.H., McCoy K.L., Palmini R.B., Junge C.E., Lee C.J., Yepes M. (2008). Plasmin potentiates synaptic N-methyl-D-aspartate receptor function in hippocampal neurons through activation of protease-activated receptor-1. J. Biol. Chem..

[59] Park H., Han K., Seo J., Lee J., Dravid S.M., Woo J., Chun H., Cho S., Bae J.Y., An H. (2015). Channel-mediated astrocytic glutamate modulates hippocampal synaptic plasticity by activating postsynaptic NMDA receptors. Mol. Brain.

[60] Maggio N., Shavit E., Chapman J., Segal M. (2008). Thrombin induces long-term potentiation of reactivity to afferent stimulation and facilitates epileptic seizures in rat hippocampal slices: Toward understanding the functional consequences of cerebrovascular insults. J. Neurosci..

[61] Almonte A.G., Qadri L.H., Sultan F.A., Watson J.A., Mount D.J., Rumbaugh G., Sweatt J.D. (2013). Protease-activated receptor-1 modulates hippocampal memory formation and synaptic plasticity. J. Neurochem..

[62] Sweeney A.M., Fleming K.E., McCauley J.P., Rodriguez M.F., Martin E.T., Sousa A.A., Leapman R.D., Scimemi A. (2017). PAR1 activation induces rapid changes in glutamate uptake and astrocyte morphology. Sci. Rep..

[63] Vance K.M., Rogers R.C., Hermann G.E. (2015). PAR1-activated astrocytes in the nucleus of the solitary tract stimulate adjacent neurons via NMDA receptors. J. Neurosci..

[64] Huda R., Chang Z., Do J., McCrimmon D.R., Martina M. (2018). Activation of astrocytic PAR1 receptors in the rat nucleus of the solitary tract regulates breathing through modulation of presynaptic TRPV1. J. Physiol..

[65] Fujita T., Liu T., Nakatsuka T., Kumamoto E. (2009). Proteinase-activated receptor-1 activation presynaptically enhances spontaneous glutamatergic excitatory transmission in adult rat substantia gelatinosa neurons. J. Neurophysiol..

[66] Price R., Ferrari E., Gardoni F., Mercuri N.B., Ledonne A. (2020). Protease-activated receptor 1 (PAR1) inhibits synaptic NMDARs in mouse nigral dopaminergic neurons. Pharm. Res..

[67] Miller S., Sehati N., Romano C., Cotman C.W. (1996). Exposure of astrocytes to thrombin reduces levels of the metabotropic glutamate receptor mGluR5. J. Neurochem..

[68] Gan J., Greenwood S.M., Cobb S.R., Bushell T.J. (2011). Indirect modulation of neuronal excitability and synaptic transmission in the hippocampus by activation of proteinase-activated receptor-2. Br. J. Pharmacol..

[69] Shavit-Stein E., Artan-Furman A., Feingold E., Ben Shimon M., Itzekson-Hayosh Z., Chapman J., Vlachos A., Maggio N. (2017). Protease Activated Receptor 2 (PAR2) Induces Long-Term Depression in the Hippocampus through Transient Receptor Potential Vanilloid 4 (TRPV4). Front. Mol. Neurosci..

[70] Hashimotodani Y., Ohno-Shosaku T., Yamazaki M., Sakimura K., Kano M. (2011). Neuronal Protease-Activated Receptor 1 Drives Synaptic Retrograde Signaling Mediated by the Endocannabinoid 2-Arachidonoylglycerol. J. Neurosci..

[71] Huang Z., Tao K., Zhu H., Miao X., Wang Z., Yu W., Lu Z. (2011). Acute PAR2 activation reduces GABAergic inhibition in the spinal dorsal horn. Brain Res..

